# Differences in host immune populations between rhesus macaques and cynomolgus macaque subspecies in relation to susceptibility to *Mycobacterium tuberculosis* infection

**DOI:** 10.1038/s41598-021-87872-x

**Published:** 2021-04-23

**Authors:** Laura Sibley, Owen Daykin-Pont, Charlotte Sarfas, Jordan Pascoe, Andrew D. White, Sally Sharpe

**Affiliations:** Public Health England – Porton, National Infections Service, Porton Down, Salisbury, Wiltshire SP4 0JG UK

**Keywords:** Infectious diseases, Innate immune cells, Lymphocytes

## Abstract

Rhesus (*Macaca mulatta*) and cynomolgus (*Macaca fasicularis*) macaques of distinct genetic origin are understood to vary in susceptibility to *Mycobacterium tuberculosis*, and therefore differences in their immune systems may account for the differences in disease control. Monocyte:lymphocyte (M:L) ratio has been identified as a risk factor for *M. tuberculosis* infection and is known to vary between macaque species. We aimed to characterise the constituent monocyte and lymphocyte populations between macaque species, and profile other major immune cell subsets including: CD4^+^ and CD8^+^ T-cells, NK-cells, B-cells, monocyte subsets and myeloid dendritic cells. We found immune cell subsets to vary significantly between macaque species. Frequencies of CD4^+^ and CD8^+^ T-cells and the CD4:CD8 ratio showed significant separation between species, while myeloid dendritic cells best associated macaque populations by *M. tuberculosis* susceptibility. A more comprehensive understanding of the immune parameters between macaque species may contribute to the identification of new biomarkers and correlates of protection.

## Introduction

Non-human primates are widely used in infectious disease research because of the similarity in immune system and physiology to humans. Several genetically distinct populations within different macaque species are available for research use, and which population/species is chosen may depend on several factors; for example, their suitability for certain infectious diseases, and ease of availability.

At Public Health England (PHE), four types of macaque have been used in Tuberculosis (TB) research; rhesus macaques (*Macacca mulatta*) (RM) of Indian genotype and cynomolgus macaques (*Macacca fasicularis*) of Mauritian (MCM) or Asian (Indonesian (ICM) and Chinese (CCM)) genotype. Macaques show a spectrum of TB disease, similar to humans^1,2^, but it is well known that the populations have differences in susceptibilities to TB disease when infected with *Mycobacterium tuberculosis* (*M. tb*); MCM and RM are more susceptible to TB than CCM^3^ and ICM^4^.

The reasons behind differences in the populations are unclear, although correlates of TB risk identified in human populations may also be applicable to macaque species. For example, our previous work had shown that monocyte:lymphocyte ratio (M:L) did differ between populations. A high, or extremely low M:L has been shown in humans to associated with risk to development of TB^5^, and in our research we have shown that RM and MCM have significantly higher M:L ratios than CCM^6^.

To further investigate differences in the host immune systems of macaque populations, a flow cytometric immunophenotyping assay was developed to compare T-cell, NK cell and monocyte subsets between populations as reports suggest that monocyte subsets and NK cells have the potential to bias the immune system to influence susceptibility to TB^5,7^.

The aim of this study was to characterise and compare these different host factors across rhesus and cynomolgus macaque species and between cynomolgus macaques of distinct genetic origin, and to identify differences between them, and determine whether these may have a role in defining their susceptibility to TB through a retrospective analysis of data from TB infection studies.

## Results

### Immune cell differences between genetically distinct macaque populations as measured with the haematology analyser

**Figure 1:**
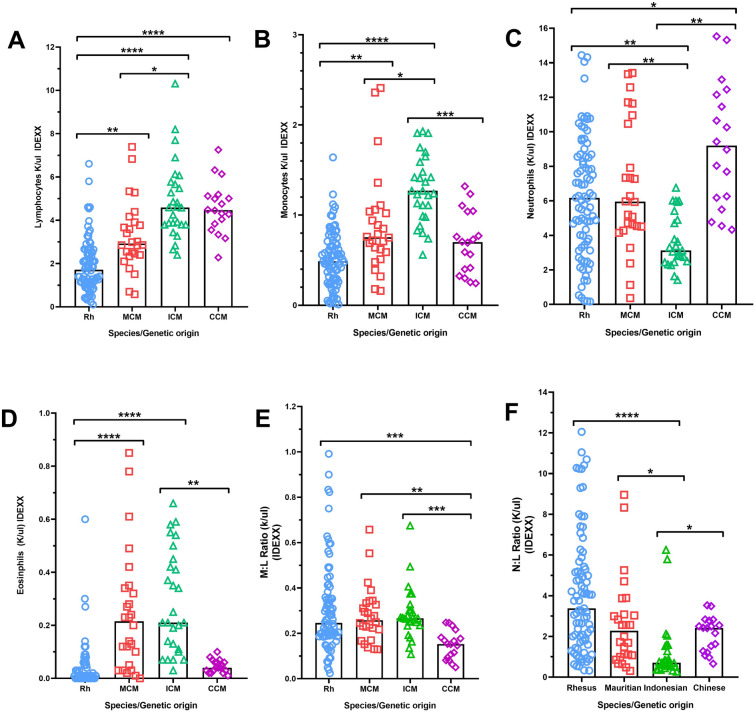
Comparison of immune cell populations and ratios between macaques of distinct genetic origin, as measured using IDEXX ProCyte Haematology analyser. (**A**) Lymphocytes, (**B**) Monocytes, (**C**) Neutrophils, (**D**) Eosinophils, (**E**) M:L, (**F**) N:L. Cell populations expressed in absolute counts K/µL. Ratios calculated from cell populations expressed in absolute counts (K/µl). Each data point represents an individual animal: Rhesus = Blue (n = 84), Mauritian = Red (n = 26), Indonesian = Green (n = 27), Chinese = Purple (n = 18), bars show the group median. Kruskall–Wallis tests with Dunn’s multiple comparisons were applied, as not all groups passed Shapiro–Wilk test for normality (*p* = 0*.*05. *p < (or equal) 0.05, **p < 0.01 and ***p < 0.001.

Differences between immune cell populations were observed between the species (Fig. 1). RM had significantly lower numbers of lymphocytes than all cynomolgus macaque populations (ICM (*p* < 0*.*001), CCM (*p* < 0.001) and MCM (*p* = 0.001) (Fig. 1A). The number of lymphocytes counted in the ICM was also significantly higher than in the MCM population (*p* = 0.034) (Fig. 1A).

RM also had the fewest monocytes (Fig. 1B), with levels significantly lower than those measured in ICM (*p* < 0.001), and MCM (*p* = 0.004). The ICM population showed a significantly higher monocyte count than both other cynomolgus populations (MCM *p* = 0*.*011, CCM *p* = 0*.*001).

ICM had significantly fewer neutrophils than all other populations (CCM (*p* < 0*.*001). RM (*p* = 0*.*002) and MCM (*p* = 0*.*004)) (Fig. 1C). The CCM population also displayed significantly higher neutrophil counts compared to RM (*p* = 0*.*025). The ICM population displayed the least variation in neutrophil count (standard deviation (SD) = 1.55); with RM, MCM and CCM displaying considerable spread within each population (SD RM = 3.45, ICM = 3.72, CCM = 3.60).

RM and CCM had fewer eosinophils than ICM and MCM (Fig. 1D); RM had significantly fewer eosinophils than both MCM and ICM (both *p* < 0*.*001) and CCM had significantly fewer eosinophils than ICM (*p* = 0*.*002), but not significantly lower than RM or MCM (Fig. 1D).

CCM had a signifcantly lower M:L than RM (*p* = 0.0001), MCM (*p* = 0*.*0010), and ICM (*p* = 0*.*001) (Fig. 1E). However, none of the other differences in M:L reached significance. The spread in values was greatest in RM (standard deviation (SD) = 3.31, MCM = 2.20), ICM = 1.47, CCM = 0.91), although this may be due to the large sample size assessed (n = 84). ICM had significantly lower neutrophil:lymphocyte ratio (N:L) compared with RM (*p* < 0*.*001), MCM (*p* = 0*.*020) and CCM (*p* = 0*.*043) (Fig. 1F). No other significant differences were observed in N:L between macaque sub-species.

### Cell frequencies as measured using immunophenotyping of PBMCs

To look in more detail at cell populations and subsets, cryopreserved PBMCs from all macaque populations were used to characterise the key immune cell populations.

**Figure 2:**
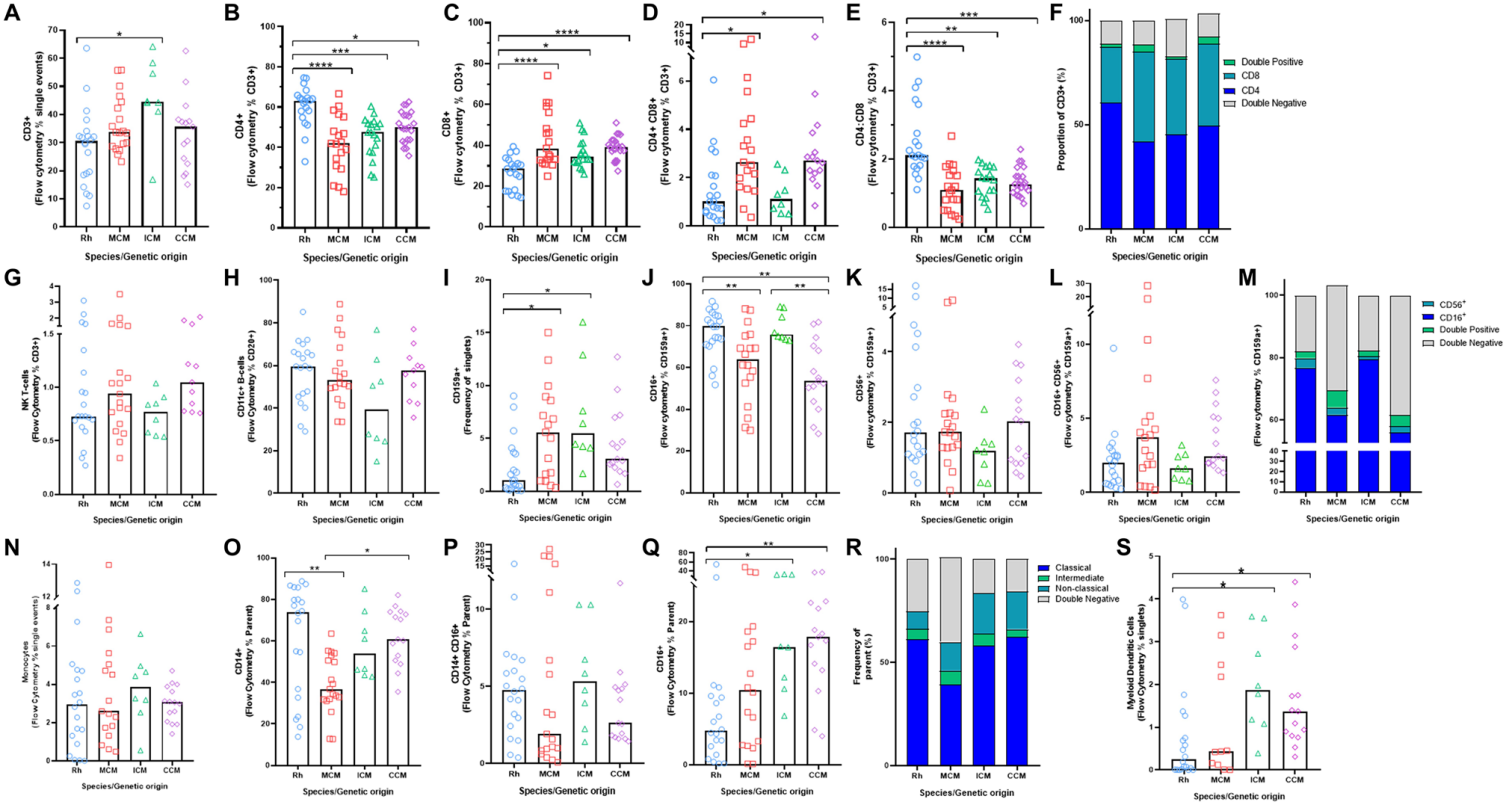
Cell populations in PBMCs determined by flow cytometric analysis in rhesus and cynomolgus macaques. (**A**) CD3^+^ lymphocytes, (**B**) CD4 + T-cells, (**C**) CD8 + T-cells, (**D**) CD4 + CD8 + DP T-cells, (**E**) CD4:CD8 ratio, (**F**) proportions of CD4^+^, CD8^+^ and CD4^+^CD8^+^ lymphocytes, (**G**) NK T-cells, (**H**) CD11c^+^ B-cells, (**I**) CD159^+^ NK cells, (**J**) CD16 + NK cells, (**K**) CD56 + NK cells, (**L**) CD16 + CD56 + DP NK cells, (**M**) proportions of cytotoxic (CD16 + CD56-), intermediate (CD16^+^ CD56^+^) and immunomodulatory (CD16^−^ CD56^+^) NK cells, (**N**) Monocytes, (**O**) CD14 + monocytes, (**P**) CD16 + monocytes, (**Q**) CD14 + CD16 + monocytes, (**R**) proportions of classical (CD14^+^ CD16^−^), intermediate (CD14^+^ CD16^+^) and non-classical (CD14^−^ CD16^+^) monocytes, (**S**) mDCs. Each data point represents an individual animal. Bars represent mean values in graphs F, M and R. Group numbers varied between comparisons, as previous immunophenotyping data for ICM and CCM was used to bolster comparisons: Rhesus (Blue) n = 20 and MCM (Red) n = 19. For lymphocyte comparisons ICM (Green) n = 18 and CCM (Purple) n = 22. For comparisons monocytes and NK cells, ICM (Green) n = 8 and CCM (Purple) n = 15. Kruskall-Wallis tests with Dunn’s multiple comparisons were applied, *p = 0.05, **p < 0.01 and ***p < 0.001.

The ICM population had a significantly higher frequency of CD3^+^ T-lymphocytes when compared to RM (*p* = 0*.*013) (Fig. 2A) (gating strategy in Supplementary Fig. S1). The frequency of CD4^+^ T-lymphocytes was significantly higher in RM compared to MCM (*p* < 0*.*001), ICM (*p* = 0.001) and CCM (*p* = 0.016) (Fig. 2B), whereas the CD8^+^ frequency was significantly lower in RM in comparison to MCM (*p *< *− *0.0001), ICM (*p* = 0.0155) and CCM (*p* ≤ 0.0001) (Fig. 2C). CD4^+^ CD8^+^ double positive (DP) T-cells were lower in RM than all cynomolgus populations, significantly so in comparison to both MCM and CCM (both *p* ≤ 0.0001) (Fig. 2D). The CD4:CD8 ratio was significantly higher in RM compared with all cynomolgus populations [MCM (*p* < 0.001), ICM (*p* = 0.001) and CCM (*p* = 0.001)] (Fig. 2E). Overall, the proportion of CD4^+^ and CD8^+^ subsets varied between groups (Fig. 2F), with MCM having the highest proportion of CD8^+^ and RM having the highest proportions of CD4^+^ T-cells. Other lymphocyte cell types including NK T-cells and antigen presenting CD11c^+^ B-cells were not found to significantly differ between macaque species (Fig. 2G,H).

Differences in NK cell frequency (CD159a^+^) were apparent between rhesus and cynomolgus species (Fig. 2I) with fewer NK cells measured in RM relative to all cynomolgus subspecies. This result was significant when compared to MCM (*p* = 0.032) and ICM (*p* = 0.010) (Fig. 2I) (gating strategy in Supplementary Fig. S1). Natural Killer (NK) cell populations are typically subdivided into cytotoxic and immunomodulatory phenotypes based on expression of the surface markers CD16 (cytotoxic) and CD56 (immunomodulatory)^8^. Cytotoxic (CD16^+^ CD56^−^) NK-cells were highest in RM with proportions significantly higher than MCM (*p* = 0.0421) and CCM (*p* = 0.002)*.* Similarly, cytotoxic NK-cells were higher in ICM than in CCM (*p* = 0.006) (Fig. 2J). The frequencies of CD16^−^ CD56^+^ and DP CD16^+^ CD56^+^ NK cells were not significantly different between populations (Fig. 2K,L), but there was a trend for MCM have a higher frequency of DP NK cells. Overall, the NK subsets measured in MCM and CCM were most similar as both had relatively high proportions of CD56^+^ and DP NK cell populations in comparison to RM, and ICM in which the fewest CD56 expressing NK-cells were detected (Fig. 2M).

The overall frequency of monocytes did not significantly differ between populations (Fig. 2N) but significant differences in the proportions of different monocyte phenotypes were observed. RM possessed the highest frequency of CD14^+^ monocytes (Fig. 2O) (gating strategy shown in Supplementary Fig. S2). MCM had a considerably lower proportion of CD14^+^ monocytes compared to the RM (*p* = 0.004) and CCM (*p* = 0.014) populations. Significant differences were not seen in frequencies of the CD14^+^CD16^+^ (Intermediate) population (Fig. 2P). ICM and CCM displayed the highest frequencies of the CD14^−^ CD16^+^ monocyte (Fig. 2Q), which were significantly higher than RM [ICM (*p* = 0.039) and CCM (*p* = 0.004)]. The total proportions of monocytes did not vary between species (Fig. 2R). Monocyte derived Dendritic Cells (mDC) were significantly higher in both ICM (*p* = 0.034) and CCM (*p* = 0.026) when compared to RM (Fig. 2S) (gating strategy in Supplementary Fig. S3).

### Relationships between cell populations and associations with TB disease

**Figure 3:**
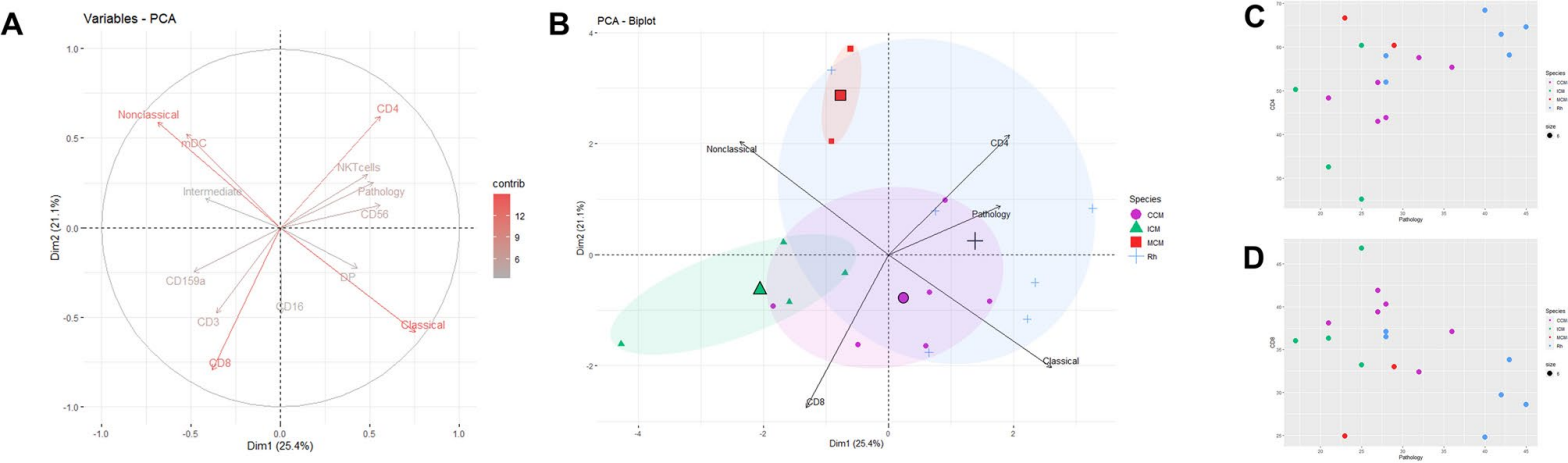
Principle component analysis of immune parameters between macaque groups using the immunophenotyping data and correlations with pathology scores. (**A**) PCA loading plot of immune parameters, with a scale for relative contribution of each component to the model. Red = highest to grey = lowest, (**B**) PCA biplot showing how the macaque populations cluster towards the variables that contribute the most to the variation in the data. Largest of each shape indicates the mean of each population with smaller shapes indicating the outliers. Blue circles = CCM, yellow triangles = ICM, grey squares = MCM, red crosses = RM (**C**) Correlation of CD4 + T-cells with pathology scores following TB infection, with each population indicated by a different colour, red = CCM, green = ICM, blue = MCM and red = RM (**D**) Correlation of CD8 + T-cells with pathology scores following TB infection, with each population indicated by a different colour, red = CCM, green = ICM, blue = MCM and red = RM.

Principle Component Analysis (PCA) was applied to immunophenotyping datasets as a multivariate analysis technique for the identification of cellular immune compartment variables that differentiated between macaque species and sub-species (Fig. 3).

Along dimension 1, the factors that account for 25.4% (eigenvalue of 3.3) of the variance in the data were classical and non-classical monocytes, pathology-based scores and CD4^+^ T-cells, with CD4^+^ T-cells clustering with pathology scores. Dimension 2, which accounted for 21.1% of the variance (eigenvalue of 2.75) were affected mostly by CD8^+^ T-cells, CD16^+^ Classical monocytes and CD4^+^ T-cells (Fig. 3A and Supplementary data Fig. S4 and Supplementary data Table S1).

Looking at the distribution of different populations of macaques, we can see that each population of macaques cluster separately, with RM having the largest variation and overlapping with MCM and CCM. ICM and RM show little overlap, and there was also little overlap between MCM and CCM. Non-classical monocytes and CD8^+^ T-cells seem to distinguish ICM from RM, and CD8^+^ and classical monocytes from CCM and MCM (Fig. 3B).

As T-cells appeared to be the most discriminatory between populations, we analysed the relationship between these cells and pathology score, calculated as described by Sharpe et al.^9^. This was used to investigate the potential for a common relationship between T-cells prior to infection and pathology score after infection with *M. tb*. We found that the CD4^+^ T-cells at baseline did correlate with an increased pathology score (*r* = 0.527*, p* = 0.025) (Fig. 3C). There was a non-significant trend for CD8^+^ T-cells to negatively correlate with pathology score (*r* = 0.445*, p* = 0.064) (Fig. 3D). Classical and non-classical monocytes were not found to correlate with pathology score (*r* = 0.270*, p* = 0.278* and r* = *− *0.111*, p* = 0.661 respectively).

## Discussion

In this study, two approaches were taken to characterise, enumerate and compare the levels of white cell populations in the blood of four genetically distinct macaque populations. Although there are limited reports comparing the immune cells of humans with those in certain macaque populations^10,11^ to our knowledge, this is the first time a direct comparison of four genetically distinct macaque populations has been performed using the same assays.

Evaluation of the cellular composition of anti-coagulated blood using a haematology analyser enables an unbiased analysis of cell population frequency and number per ml of blood for all types of cells present. Conversely, data originating from PBMC samples relates to mononuclear cell populations only and is proportional, but the main advantages of flow cytometric immunophenotyping is that it can generate more detailed data and can be applied to archived samples enabling retrospective interrogation of materials.

The haematology analyser derived data set demonstrated that lymphocytes and monocyte counts were different between rhesus and cynomolgus macaque species, whereas there was little difference between the genetically distinct cynomolgus populations. Separating the populations into TB disease susceptible (RM and MCM) and less susceptible (ICM and CCM) as suggested by the literature^4,12^, it was only lymphocyte levels that were different between these groupings, and suggests that higher numbers of lymphocytes are important for protection against *M. tb*. Eosinophil and neutrophils counts were different between groups, but the differences were not common between the susceptible and less susceptible groups and so, it is perhaps unlikely that basal eosinophils and neutrophil numbers have a significant bearing on susceptibility to TB disease.

Immunophenotyping studies of PBMC were used to evaluate the subtypes of the lymphocytes and monocytes present. When the least susceptible populations (ICM and CCM) were grouped and compared to the susceptible populations (RM and MCM), key differences were identified in the frequency of mDC. The CD4:CD8: ratio also differed between RM and cynomolgus macaques.

The availability of fewer samples from some macaque populations compared to others limited the ability to interpret differences between populations from the PCA analysis. However, by evaluation of the all animals representing a group with a variety of outcomes following *M. tb* infection, the impact of different cell populations in general on TB susceptibility can be evaluated. The frequency of CD4^+^ T-cells was identified using PCA to group the macaque populations generally with some overlap, and the frequency of CD4^+^ T-cells correlated with TB-induced disease burden measured using a pathology-based scoring system. There are also many subtypes of CD4^+^ T-cells to be considered, and this analysis may be too simplistic, and subtypes, functionality and activation status also need to be considered in future analysis. The difference in lymphocyte number between macaque populations revealed by haematology analysis, when taken with the relationship identified by PCA for CD4^+^ T cells suggest both lymphocyte number and subtype have a role in TB susceptibility; such that a higher number of lymphocytes but a lower proportion of CD4 + T-cells may help disease control.

Frequency of CD8^+^ T-cells and CD16^+^ NK cells contributed to the PCA second dimension, highlighting a potential difference in cells that have cytolytic roles between primate populations. We saw a non-significant correlation between higher numbers of CD8^+^ cells before infection that correlated with lower pathology scores. In CD8^+^ depletion studies in primates, CD8^+^ T-cells have been shown to be important in the control of infection^13^. The CD4:CD8 ratios defined in ICM and CCM showed a balance in CD4^+^ and CD8^+^ T-cells, whereas in MCM a skew towards the CD8 population was found, in line with the previous report from Zitsman et al.^14^, whereas the populations in RM were more biased towards CD4. A low CD4:CD8 ratio has been found to be a predictor TB in HIV patients^15^ so as MCM do have the lowest CD4:CD8 ratio, this could be a contributing risk factor in their susceptibility to TB.

NK cell transcripts were found to be lower in CMV + infants, that went on to develop TB^7^ suggesting a link between NK cells and TB susceptibility. A study comparing NK subsets between persons from a TB endemic country with TB naïve persons showed that there was little difference in the frequency of cytolytic NK cells, but that those NK cells had different reactivity and functional capacity^16^. Therefore, in this study, the NK subtype proportions were most similar between MCM and CCM which have very different susceptibilities to TB so further investigation into the functionality of the NK cells is required to determine whether there are differences in their capacity to react to TB and influence disease progression.

Monocytes contributed to the variance in the first dimension in the PCA, and there was a difference in the proportion of CD14^+^ classical monocytes between rhesus and cynomolgus macaques, but they did not correlate with pathology. Dijkman et al. saw differences in monocyte subtypes and cytokine production post-infection with TB between rhesus and cynomolgus macaques^17^ and so looking post-infection at whether there are differences between populations in how they respond to infection that relates to their basal subtypes would be something to examine further in future work.

Antigen presenting cells (APCs) such as mDCs are a key component of T-cell activation, and this population was present at significantly higher frequencies in the macaque populations that are less susceptible to TB. Efficient priming of T-cells is considered to be key in protecting against TB and TB modulates DC activity by delaying their ability to migrate to the lymph nodes, hampering the formation of an effective immune response and giving the TB infection time to establish^18^. Furthermore DCs have been found to be present at lower levels in patients with TB^19^. Having a higher number of mDCs has the potential to confer an advantage by increasing the likelihood of migration to the lymph nodes and increasing the interactions with T-cells to promote an early immune response to infection.

Overall, these studies have revealed differences in the cellular composition of peripheral blood in four genetically distinct macaque populations, and particularly between rhesus and cynomolgus macaques in terms of lymphocyte populations. The concordance of findings from haematology analyser-based and flow cytometry-based measurements, supports the concept that there are fundamental differences in the makeup of the immune systems of these species. Others have noted that macaques vary genetically substantially between geographical locations^20^, and recommend caution when comparing data from different models for the same diseases as contributing factors could obscure risk factor-disease associations, or lead to artificial associations. Therefore, it is important to understand the genetic background of the animals used in studies, together with the potential implications that any consequent constitutive differences between populations may have on the experimental outcome. Characterisation of macaque populations provides the opportunity to select populations with desirable characteristics for specific studies so differences can be exploited to further understand the factors required to promote a successful immune system.

## Methods

### Information on use of animal tissues

This study was a retrospective analysis of data generated from samples collected from animals housed within the PHE-managed macaque breeding units and from macaques enrolled in previous studies at PHE Porton. RM and cynomolgus macaques were obtained from established UK Home Office approved breeding colonies in the United Kingdom (ICM, MCM, RM) and China (CCM). Genetic analysis of macaques from the UK colonies has previously confirmed the rhesus macaques to be of the Indian genotype and cynomolgus macaques of Mauritian^21^ or Indonesian^22^ genotypes. The numbers of each species used for each type of analysis is outlined in Table 1. Information on housing and procedures have been described elsewhere^23^.

**Table 1:** Number of samples from each population for each type of analysis.

	RM	MCM	ICM	CCM
Haematology analyser	84	26	27	18
Flow cytometry	20	19	8	15
Flow cytometry M:L	10	6	6	6
TB infection studies	6	2	4	6

All animals that had taken part in TB infection studies were challenged with *M. tb* strain Erdman K 01 (BEI resources) at the estimated retained doses shown in Table 2 using the method of aerosol infection which has been previously reported^4,23–26^. A description of the necropsy procedures, pathology and bacteriology processes have been described for the RM; S36, S40, S51, S50 and S33^23^, MCM^12^ and CCM (submitted^27^) and similar procedures were used for all studies. A description of the pathology scoring system is reported by Sharpe *et al*^9^.

**Table 2:** Estimated retained doses of *M. tb*, study lengths and end points of animals that took part in TB infection studies.

ID	Species	Estimated retained dose (CFU)	Study length (weeks)	End point (weeks)
D19	RM	75	12	5
S36	RM	100	17	7
S40	RM	100	17	15
S51	RM	100	17	7
S50	RM	100	17	17
S33	RM	100	17	7
0385	CCM	1000	28	28
0803	CCM	1000	28	28
1027	CCM	1000	28	10
4389	CCM	1000	28	5
8979	CCM	1000	28	28
9623	CCM	1000	28	7
802HAHA	ICM	75	12	12
044HAFB	ICM	75	12	12
978AN	ICM	30	12	12
548FBGA	ICM	30	12	11
M054E	MCM	43	13	13
M064D	MCM	35	13	13

### IDEXX ProCyte DX

Blood samples anti-coagulated with EDTA (1.8 mg/ml blood) or heparin, (both BD Biosciences, USA). All ProCyte DX were analysed using the IDEXX ProCyte DX Haematology analyser (IDEXX, USA). Results are expressed as absolute counts (K/µl), or ratios thereof.

### PBMC Isolation and resuscitation

Peripheral blood mononuclear cell (PBMC) samples were isolated using standard methods^28^. The density gradient material used for PBMC isolation was dependent on the macaque species; Ficoll Histopaque (GE Healthcare, USA) for rhesus macaques or Percoll (Sigma-Aldrich, UK) for cynomolgus macaques. Samples were stored at − 180 °C in isothermal tanks prior to analysis.

Upon resuscitation for analysis, samples were washed twice with RPMI media (Sigma-Aldrich, UK) supplemented with 10% foetal calf serum (FCS), with added DNase (1 Unit ml^−1^) (Sigma-Aldrich, UK) by centrifugation at 400 g for 5 min. The samples were rested for either 2 h, or overnight, at 37 °C, 5% CO_2_.

### Flow cytometric staining

Following resting of cells, adherent cells were washed from the tube by addition of 2 mM EDTA (Sigma-Aldrich, UK) and gently agitated for 15 min. A viable cell count was performed, and 1.5 × 10^6^ cells per animal were used for flow cytometric staining. LIVE/DEAD Fixable Dead Stain Kit Violet (Invitrogen, UK) was used according to manufacturer protocol, prior to application of other antibodies to reduce background staining; and was incubated for 30-min. Cells were then washed by centrifugation at 400 g for 5 min and resuspended in PBS. Staining with the full panel of antibodies was applied and incubated for 30 min according to the information in Table 2. Lymphocytes and monocytes were initially gated using forward scatter (FSC) and side scatter (SSC). Lymphocytes were then determined using Live/Dead and CD20^−^ CD3^+^ staining and CD4^+^ and CD8^+^ staining from the CD20^−^ CD3^+^ population (Supplementary data Fig. S2). NK T-cells were taken from the CD20^−^ CD3^+^ population and were CD16^+^. CD11c B-cells were CD3^−^, CD20^+^ and CD11c^+^ (Supplementary data Fig. S2). NK Cells were CD3^−^, CD8^+^ HLA-DR^−^ and then CD159a^+^. The subsets of NK cells were taken from the CD159a^+^ population and were defined as either CD16^+^ CD56^−^ (cytotoxic), CD16^+^ CD56^+^ (intermediate) or CD16^−^ CD56^+^ (immunomodulatory) (Supplementary data Fig. S2). From the initial monocyte gate, monocytes were characterised by being CD3^−^ and CD20^−^, then CD14^+/−^ and HLA-DR^+^ and subsets were defined using CD14^+^ CD16^−^ (classical), CD14^+^ CD16^+^ (intermediate) and CD14^−^ CD16^+^ (non-classical) (Supplementary data Fig. S3). The mDCs were defined as CD14^−^ CD3^−^, and then CD8^−^ CD20^−^, followed by CD159a^−^ gating, HLA-DR^+^ gating and then characterised as being CD16^+^ and CD11c^+^ (Supplementary data Fig. S4). Following antibody labelling cells were washed twice in PBS by centrifugation at 400 g for 5 min, resuspended in 4% paraformaldehyde solution and rested for a minimum of 20 min, prior to flow cytometric acquisition using LSRII Fortessa flow cytometer (BD Biosciences, Oxford, UK) (Table 3).

**Table 3:** Antibody panel.

Antigens	CD3	CD4	CD8	CD11c	CD14	CD16	CD20	CD56	CD159a	HLA-DR	Live/Dead
Lymphocyte	CD3	CD4	CD8	–	–	–	–	–	–	–	L/D
NK-cell	–	–	CD8	–	–	CD16	–	CD56	CD159a	Dim/–	L/D
Monocyte	–	–	–	–	CD14	CD16	–	–	–	HLA-DR	L/D
mDC	–	–	–	CD11c	–	CD16	–	–	–	HLA-DR	L/D
B-cell	–	–	–	CD11c	–	–	CD20	–	–	–	L/D
NKT Cell	CD3	–	CD8	–	–	CD16	–	CD56	–	–	L/D
Fluorochromes	AF700	PerCP-Cy5.5	APC-Fire750	PE	APC	BV786	PE-Dazzle	BV605	PE-Cy7	BUV395	Violet
Clone	FN-18	OKT4	SK1	3.9	M5E2	3G8	2H7	MY31	Z199	G46-6	N/A
Manufacturer	BD	Biolegend	Biolegend	Biolegend	Biolegend	Biolegend	Biolegend	BD	Beckman Coulter	BD	Invitrogen

### Data analysis

IDEXX data was analysed using GraphPad Prism V8.0.1 (GraphPad Software Inc, USA) and the Kruskal–Wallis test for multiple comparisons was applied to data sets. Flow cytometry data was analysed using FlowJo V10 (BD Biosciences, UK) and data exported for analysis using GraphPad Prism V8.01. Data was tested for normality using Shapiro–Wilk test for normality and the Kruskall-Wallis tests with Dunn’s corrections for multiple comparisons were applied to data. Principle Component Analysis (PCA) was carried out using RStudio version 3.5.3 (RStudio Inc., Boston, MA, USA).

### Ethical approval

All animal procedures and study design were approved by the Public Health England, Animal Welfare and Ethical Review Body, Porton Down, UK, and authorised under an appropriate UK Home Office project license.

## Supplementary information

Supplementary information.

## Data Availability

All data generated or analysed during this study are included in this published article (and its Supplementary Information files).
