# Supplementary material for: Differences in host immune populations between rhesus macaques and cynomolgus macaque subspecies in relation to susceptibility to Mycobacterium tuberculosis infection
Source: Sci Rep. 2021 Apr 23;11:8810. doi: 10.1038/s41598-021-87872-x (PMC8065127; doi:10.1038/s41598-021-87872-x)
Supplement: Supplementary file 1 — Supplementary information. [file 41598_2021_87872_MOESM1_ESM.docx]

**Differences in host immune populations between rhesus macaques and cynomolgus macaque subspecies, in relation to susceptibility to *Mycobacterium tuberculosis* infection**.

Laura Sibley*^1^, Owen Daykin-Pont*^1^, Charlotte Sarfas^1^, Jordan Pascoe^1^, Andrew D. White^1^, Sally Sharpe^1^.

^1^Public Health England – Porton, Salisbury, UK

Corresponding author: Laura Sibley
Address: Public Health England – Porton, Porton Down, Salisbury, Wiltshire, SP4 0JG, UK.
Telephone number: 01980 619864
e-mail address: [Laura.sibley@phe.gov.uk](mailto:Laura.sibley@phe.gov.uk)

**Supplementary data**


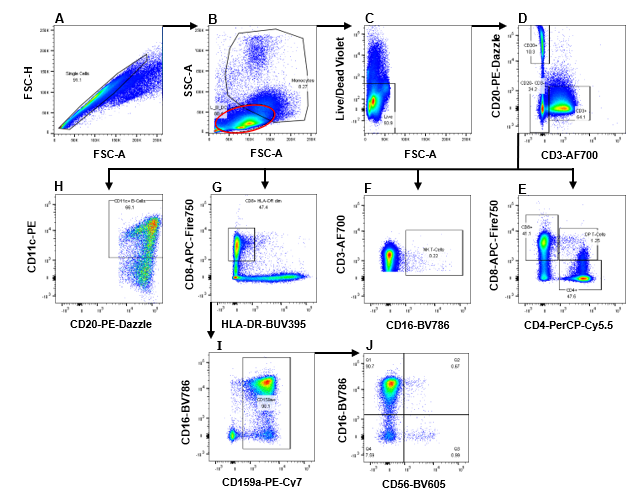


**Supplementary figure 1:** *Flow cytometric analysis of T and B-lymphocytes and NK-cells.* Selection for all cell types was preceded by A) exclusion of doublets, B) gating by FSC SSC (A) profile, and C) selection of viable cells only. **T-lymphocytes** selected as D) CD3^+^ CD20^-^, from which E) three T-lymphocyte phenotypes were described by expression of CD4 and CD8: CD4^+^, CD8^+^ and CD4^+^CD8^+^ (DP T-cells). **Natural killer cells** were selected D) as CD3^-^CD20^-^, and G) CD8^+^ HLA-DR^dim/-^. Following this, I) CD159a^+^ cells were selected, presenting J) three NK-cell phenotypes by expression of CD16 and CD56. A small population of **NK T-cells** were identified as D) CD3^-^CD20^-^, and F) CD16^+^. **B-cells** were identified as D) CD20^+^CD3^-^, from which H) a CD11c^+^ population were evaluated, with reference to appropriate fluorescence minus one controls. All plots in this figure are from the same animal (ICM) and are representative of wider population. Values presented on gates are given as frequency of parent gate.


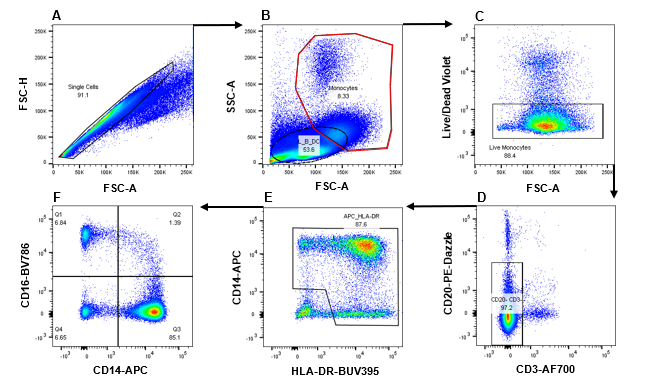


**Supplementary figure 2:** *Flow cytometric analysis of monocyte populations by differential expression of CD14 and CD16.* Monocytes were firstly gated based on FSC-A and FSC-H profiles, to exclude doublets. Monocytes were then selected for by their SSC and FSC (A) profiles, largely excluding lymphocytes. Following this: A) Dead cells, B) CD3^+^ T and CD20^+^ B-cells were excluded. PBMC samples have minimal granulocyte infiltration; any remaining infiltrates were excluded based on being HLA-DR^-^ CD14^dim^ (C). Finally, C) CD14^+^ and HLA-DR^+^ were selected for, yielding three monocyte phenotypes (F) based on their expression of CD14 and CD16: classical (CD14^+^), intermediate (CD14^+^ CD16^+^), non-classical (CD16^+^).


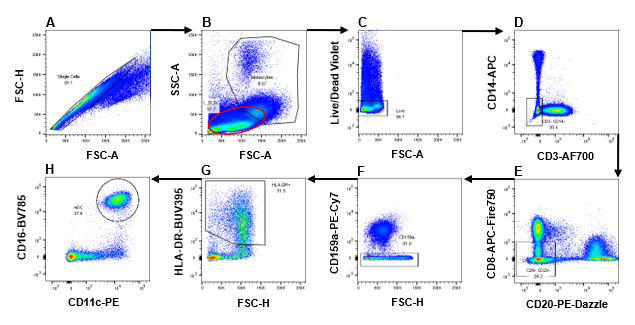


**Supplementary figure 3:** *Flow cytometric analysis of myeloid dendritic cells by expression of CD11c and CD16.* Initially, A) singlets were excluded. Myeloid dendritic cells were then gated for based B) on their FSC SSC (A) profiles, and C) viability. D) CD3^-^ CD14^-^ (Lin^-^) cells then selected for, and E) CD8^+^ and CD20^+^, as well as F) CD159a^+^ NK cells were excluded. G) HLA-DR^+^ cells were selected for, yielding H) myeloid dendritic cells as seen by co-expression of CD16 and CD11c.

**
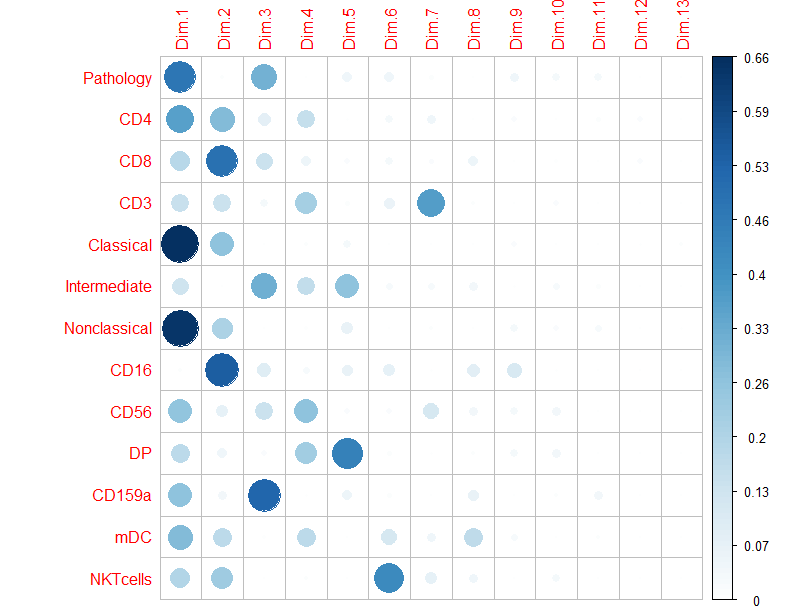
**

**Supplementary data 4.** *Variables contributing to the dimensions of the PCA analysis.* The contribution of each variable to the variance in each dimension is shown by the size of the circle and is relative to the other variables.


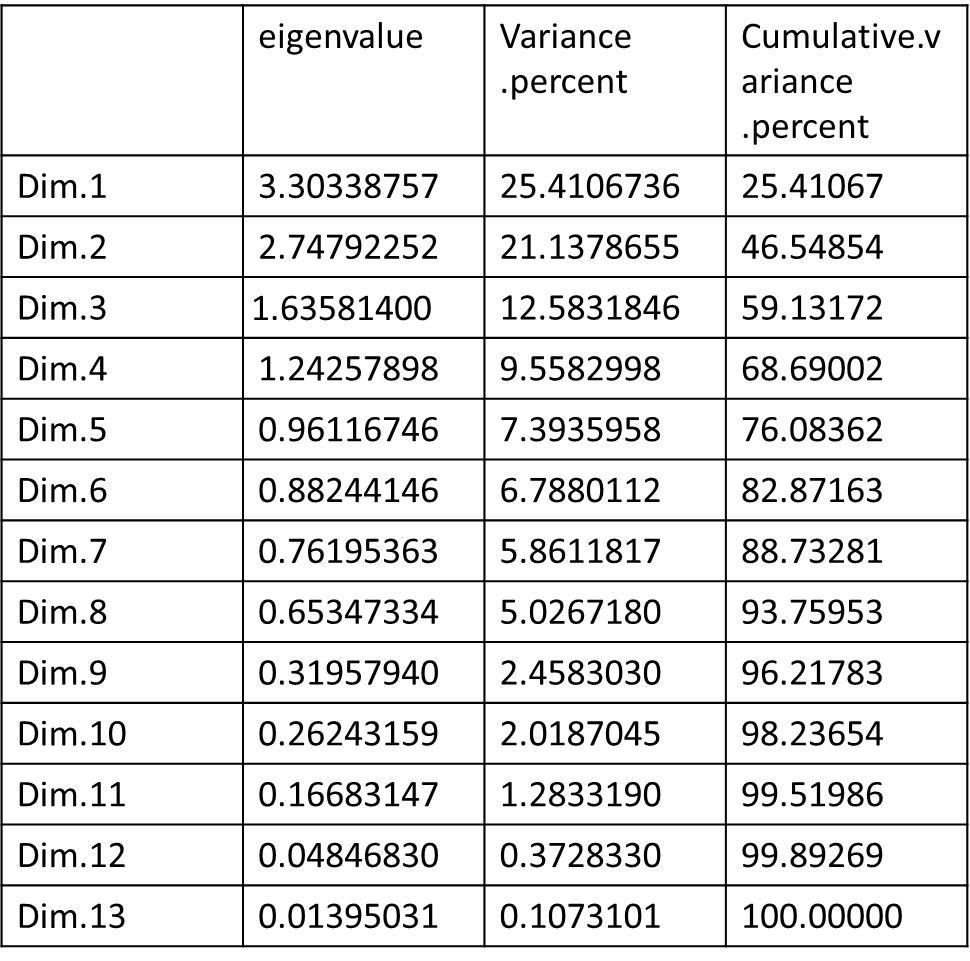


**Supplementary Table 1.** *Relative contributions of each dimension of the PCA analysis to the variance in the data.*
